# 2064. *In Vitro* Susceptibility of Recent *Mycobacterium abscessus* Isolates to Epetraborole (EBO) and Comparators by Broth Microdilution

**DOI:** 10.1093/ofid/ofad500.134

**Published:** 2023-11-27

**Authors:** Minh-Vu H Nguyen, Vinicius Calado Nogueira de Moura, Tiffany Keepers White, Charles L Daley

**Affiliations:** National Jewish Health, Denver, CO; National Jewish Health, Denver, CO; AN2 Therapeutics, Menlo Park, California; National Jewish Health, Denver, CO

## Abstract

**Background:**

*Mycobacterium abscessus* (MAB) is a highly drug-resistant nontuberculous mycobacterium (NTM) that is an important cause of pulmonary disease (PD). Treatment is limited by the lack of active oral drugs and frequent adverse reactions. EBO is a novel oral, boron-containing antimicrobial that inhibits bacterial leucyl-tRNA synthetase, an essential enzyme in protein synthesis. It has demonstrated encouraging *in vitro* and *in vivo* efficacy against MAB in small, limited preclinical studies and is currently in clinical development for the treatment of other NTM-PD. This study evaluated EBO minimal inhibitory concentrations (MIC) against a larger number of recent MAB isolates.

**Methods:**

MAB isolates collected in 2021 from the United States (n=122) from respiratory sources and in 2019-2022 from Europe (n=25) from respiratory and other sources were tested by broth microdilution according to Clinical and Laboratory Standards Institute guidelines using frozen microtiter panels manufactured by ThermoFisher against EBO and a panel of 13 antimicrobials with anti-MAB activity. MIC values were determined after 4-5 days of incubation; if an isolate was susceptible to clarithromycin (CLR) at day 5, it was reread at day 14 to assess for inducible macrolide resistance. Descriptive analyses were done on the MIC values.

**Results:**

Of the 147 MAB isolates, 101 were subspecies *abscessus*, 6 were *bolletii*, and 40 were *massiliense*. EBO MIC_50_/MIC_90_ for all isolates were 0.06/0.12 mg/L. MICs ranged from 0.03 - 0.25 mg/L and were consistent across subspecies (Table 1). MIC ranges, MIC_50_, and MIC_90_ values for all agents are summarized in Table 2. 95 isolates (64.6%) were CLR-resistant, consisting of 73 isolates with inducible and 22 with constitutive resistance. The EBO MIC_50_/MIC_90_ values for either CLR-resistant or amikacin (AMK)-resistant isolates remained 0.06/0.12 mg/L.

**Figure:**
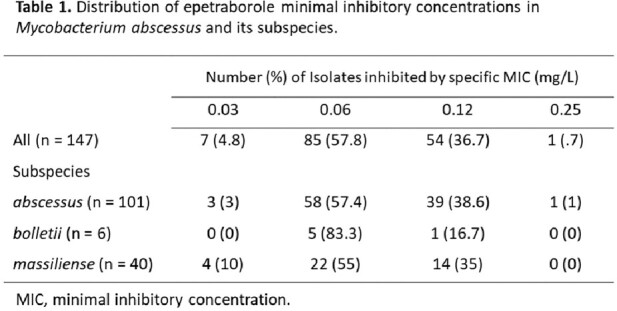

**Figure:**
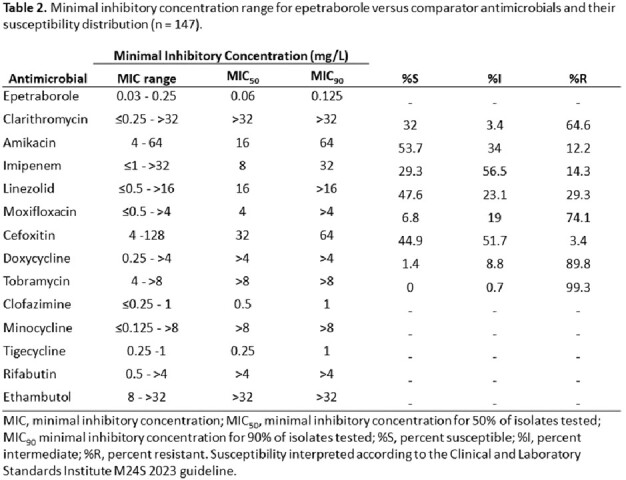

**Conclusion:**

EBO demonstrated potent *in vitro* activity against MAB with MICs from 0.03-0.25 mg/L with similar activity against all subspecies. EBO MICs remained low against MAB isolates that were resistant to other agents, including CLR and AMK. These data demonstrate EBO’s promising *in vitro* activity versus MAB regardless of subspecies or resistance to current agents and support clinical evaluation of EBO as a therapeutic option for MAB disease.

**Disclosures:**

**Tiffany Keepers White, PhD**, AN2 Therapeutics: Employee|AN2 Therapeutics: Stocks/Bonds **Charles L. Daley, MD**, AN2: Advisor/Consultant|AN2: Grant/Research Support|Aztrazeneca: Advisor/Consultant|Beyond Air: Grant/Research Support|Bill and Melinda Gates Foundation: Data Monitoring Committee|Bugworks: Grant/Research Support|Genentech: Advisor/Consultant|Hyfe: Advisor/Consultant|Insmedd: Advisor/Consultant|Insmedd: Grant/Research Support|Juvabis: Grant/Research Support|Lilly: Data Monitoring Committee|MannKind: Advisor/Consultant|Matinas: Advisor/Consultant|Otsuka: Data Monitoring Committee|Paratek: Advisor/Consultant|Paratek: Grant/Research Support|Pfizer: Advisor/Consultant|Spero: Advisor/Consultant|Zambon: Advisor/Consultant

